# Functional Recovery of Posterior Interosseous Nerve Injury Treated With a Nerve Conduit: A Case Report

**DOI:** 10.7759/cureus.83303

**Published:** 2025-05-01

**Authors:** Ken Sasaki, Toru Sasaki, Akiko Yamamoto, Teruhiko Sekiguchi, Yoshiaki Wakabayashi

**Affiliations:** 1 Department of Orthopaedic Surgery, Yokohama City Minato Red Cross Hospital, Yokohama, JPN; 2 Department of Functional Joint Anatomy, Institute of Science Tokyo, Tokyo, JPN; 3 Department of Orthopaedic and Spinal Surgery, Institute of Science Tokyo, Tokyo, JPN; 4 Department of Neurology, Yokohama City Minato Red Cross Hospital, Yokohama, JPN

**Keywords:** electromyography, motor conduction studies, motor nerve injury, nerve conduit, posterior interosseous nerve

## Abstract

In peripheral nerve injuries, autologous nerve grafting is often performed when direct anastomosis is difficult, but in cases involving sensory nerves, nerve conduits may be useful. However, there are few reports on the use of nerve conduits for motor nerve injuries. In this case, we used a nerve conduit to treat a posterior interosseous nerve injury, which is a predominantly motor nerve, and achieved relatively favorable results. This case suggests that nerve conduits could be useful for small-gap, thin motor nerve defects, especially when the injury is close to the neuromuscular junction.

## Introduction

Peripheral nerve injuries are frequently encountered in clinical practice, and their treatment largely depends on the severity and type of the injury. It is crucial to develop an optimal treatment strategy based on the extent of the damage. In mild cases where nerve continuity is preserved, spontaneous recovery can often be expected with conservative management. For complete nerve transections, tension-free end-to-end microsurgical repair is considered the gold standard. When a direct repair is not feasible due to a significant nerve gap, autologous nerve grafting remains the most reliable method for bridging the defect. Common donor sites include sensory nerves such as the sural nerve. Nerve conduits have been developed as alternatives to autografts and are especially effective for sensory nerve defects less than 3 cm in length. These conduits facilitate axonal regeneration by guiding the regrowth across the gap. Processed nerve allografts have also gained increasing attention in recent years, particularly for longer sensory nerve gaps. The choice among these treatment options depends on several factors, including the type of nerve involved (motor or sensory), the length of the defect, the time since the injury, and the overall condition of the patient. The efficacy of nerve conduits for sensory nerves, such as digital nerves, has been reported [1,2]. However, for motor nerve repair, nerve conduits are generally considered inferior to autologous nerve grafting [3], likely due to the complexity of motor nerve regeneration, which involves precise reinnervation and long-distance axonal regeneration. Given these challenges, we report a case of traumatic posterior interosseous nerve rupture successfully treated with a nerve conduit, demonstrating functional improvement.

## Case presentation

**Figure 1:**
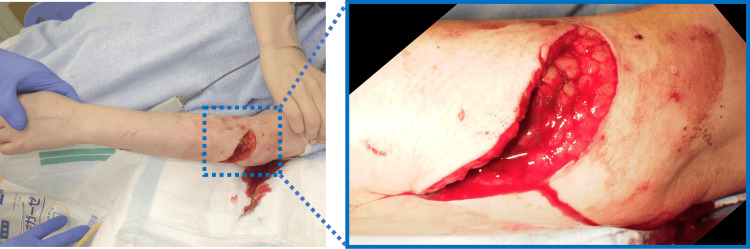
Photograph at the time of injury A laceration on the proximal dorsal side of the left forearm with damage observed in the extensor group.

The patient was a 44-year-old right-handed woman who sustained an injury to the extensor side of her left forearm caused by glass fragments (Figure 1). On the day of the injury, she underwent skin suturing in the emergency department. From the time of injury, she exhibited weakened wrist extension strength and an inability to extend her fingers, raising suspicion of posterior interosseous nerve injury. Two days after the injury, she underwent nerve repair surgery by the orthopedic team.

**Figure 2:**
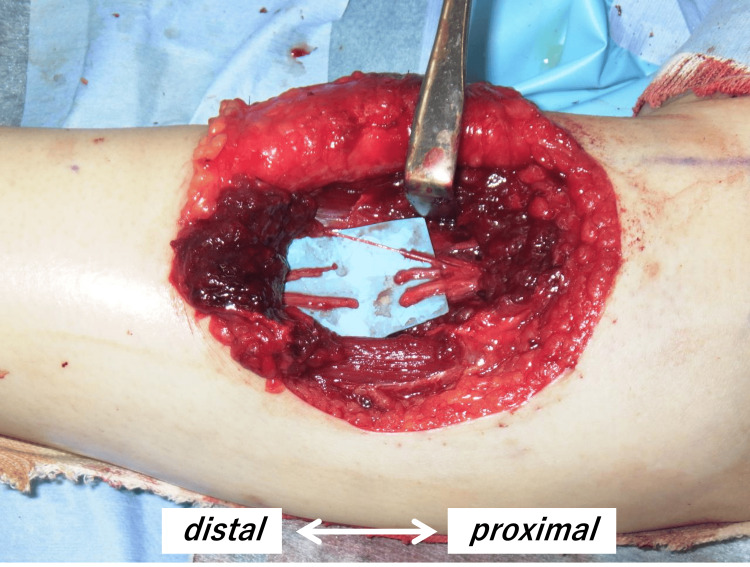
Damaged posterior interosseous nerve The epineurium was damaged, and the nerve was divided into several fascicles.

**Figure 3:**
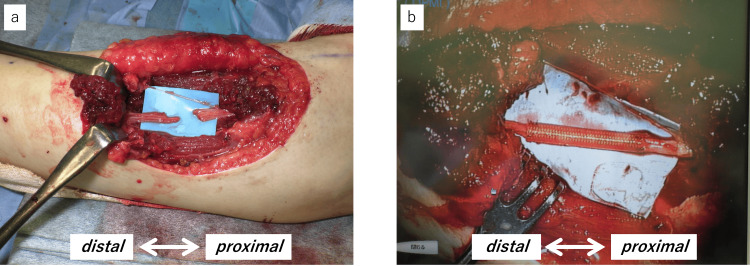
Nerve repair surgery a. Side-to-side suturing of the separated nerve fascicles, leaving a gap of approximately 2 cm. b. Nerve repair using a 3-mm diameter, 30-mm long nerve conduit.

The incision was extended distally, revealing damage to the extensor carpi radialis, extensor digitorum communis (EDC), and supinator muscle. The posterior interosseous nerve beneath the supinator muscle was found with an injured epineurium and separated into several nerve fascicles. After detaching the adhesions from the surrounding soft tissues and approximating the nerve ends as much as possible, a 2 cm gap remained due to insufficient tension-free approximation, making direct anastomosis impossible (Figure 2). Given the injury's proximity to the neuromuscular junction of the EDC and the need to avoid sacrificing healthy tissue for nerve grafting, a nerve conduit was chosen for repair. After performing side-to-side suturing of the separated nerve fascicles (Figure 3a), a 3-mm diameter, 30-mm length nerve conduit (Nerbridge® manufactured by Toyobo Co., Ltd., Japan) was applied for repair (Figure 3b). Postoperatively, the limb was immobilized with the elbow in 90 degrees of flexion and the forearm in a neutral position between pronation and supination for three weeks, followed by the use of a wrist extension brace.

**Figure 4:**
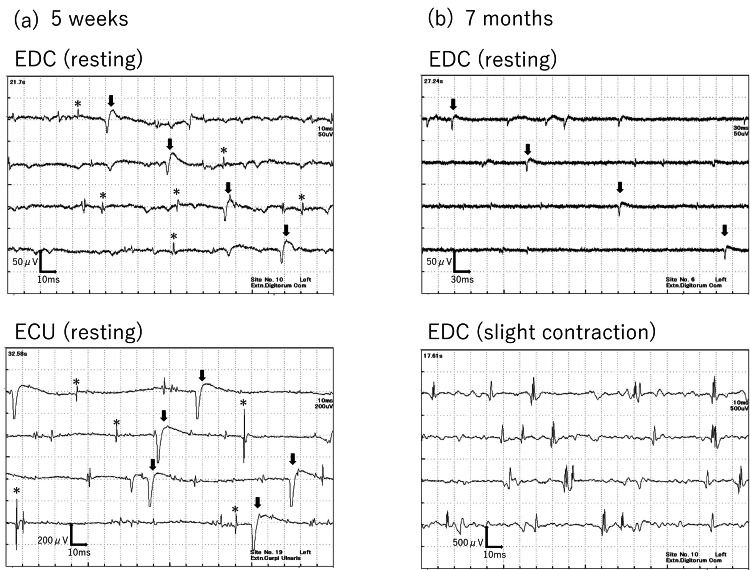
Results of needle electromyography a. At five weeks postoperatively, electromyography revealed fibrillation potentials (*) and positive sharp waves (arrow) in the extensor digitorum communis (EDC) and extensor carpi ulnaris (ECU). b. At seven months postoperatively, electromyography of the EDC showed reduced abnormal spontaneous activities and motor unit potentials, including polyphasic waves, during slight contraction.

Electromyography (EMG) performed five weeks postoperatively revealed fibrillation potentials and positive sharp waves in the EDC and extensor carpi ulnaris (ECU) muscles (Figure 4a). No motor unit potentials could be activated. At seven months postoperatively, EMG showed a reduction in spontaneous potentials at rest in the EDC, along with the appearance of motor unit potentials, including polyphasic waves during weak contraction (Figure 4b). These findings objectively indicated the process of reinnervation through electrophysiological evaluation. Clinically, wrist extensor strength improved to grade 5 on manual muscle testing (MMT) at six months postoperatively, and finger extension reached MMT grade 3 by 17 months.

**Figure 5:**
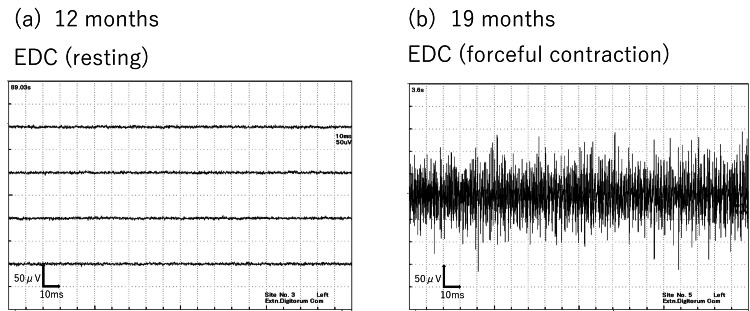
Progression of needle electromyography a. At one year postoperatively, spontaneous potentials at rest in the extensor digitorum communis (EDC) had disappeared. b. At 19 months, normalization of interference patterns during maximal contraction was confirmed.

**Figure 6:**
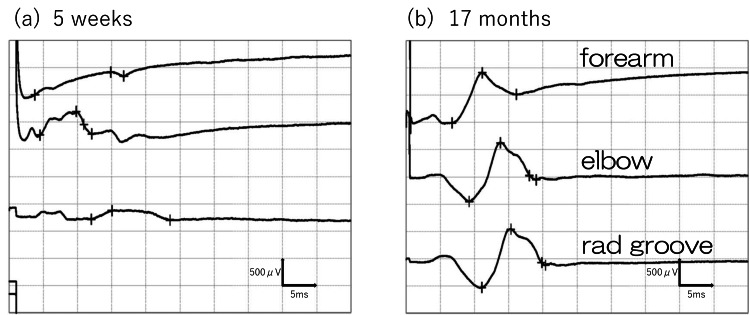
Compound muscle action potential (CMAP) of the extensor indicis proprius (EIP) a. At five weeks postoperatively, the waveform was undetectable. Note that the small CMAP, probably through the current spread to the median nerve, was recorded after the stimulation at the elbow. b. At 19 months postoperatively, the waveform became detectable.

**Figure 7:**
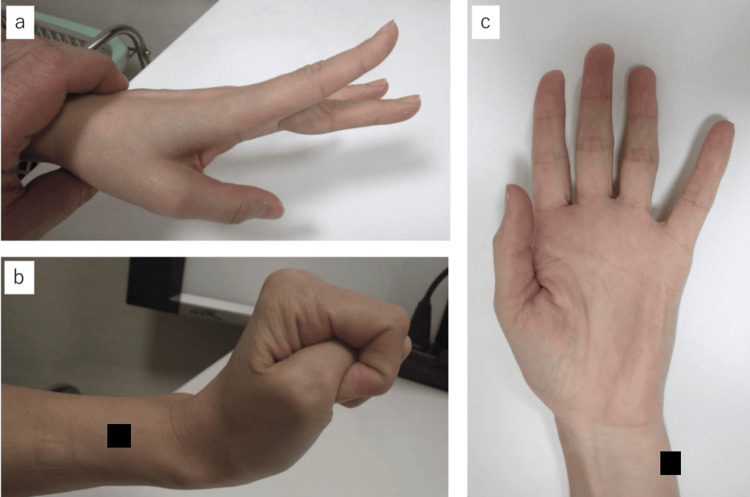
Upper limb function at 18 months postoperatively Improvements in extension of the index to little fingers and wrist dorsiflexion were observed (a, b), while extension and abduction of the thumb did not improve (c).

By 12 months postoperatively, EMG confirmed the disappearance of abnormal spontaneous activities in the EDC (Figure 5a), and at 19 months, normalization of interference patterns during maximum contraction was observed (Figure 5b). Moreover, in motor conduction studies (MCS) of the extensor indicis proprius (EIP), the compound muscle action potential (CMAP) waveform, which was almost undetectable immediately after surgery, became detectable at 19 months (Figure 6). The slight potentials may be due to direct muscle stimulation (e.g., ECR) from forearm stimulation, current spread to the median nerve at the elbow, or activation via intact radial nerve fibers from upper arm stimulation. By 18 months postoperatively, improvement in the extension of the index to small fingers and wrist dorsiflexion was achieved, with a Hand20 score of 17.5, indicating good functional recovery (Figure 7) [4]. The Hand20 score is a widely used tool in Japan for subjective hand function assessment. It evaluates hand-related pain, strength, and dexterity. The maximum score is 100 points, with lower scores indicating better hand function. The questionnaire is simple, reliable, and effective for assessing hand function, making it commonly used in both clinical and research settings. Despite persistent limitations in active thumb extension and abduction (passive movement was possible), the patient declined reconstructive surgery.

## Discussion

We performed nerve repair using a nerve conduit for a traumatic posterior interosseous nerve rupture and achieved relatively favorable results. Furthermore, the recovery process was objectively demonstrated through electrophysiological examination.

The nerve conduit consists of a bioabsorbable polyglycolic acid (PGA) tube filled with a collagen sponge, and its efficacy in treating sensory nerve injuries has been reported in the literature [1,2]. However, it has also been reported that nerve conduits are less effective for gaps larger than 3 cm or for thicker nerves [5,6]. Specifically, the outcomes for motor or mixed nerves are often poor. The reasons are as follows: (1) When the distance between the injury site and the neuromuscular junction is long, it takes significant time for nerve regeneration, leading to muscle atrophy [7]; (2) Even if regenerated axons reach the neuromuscular junction, muscle repair cannot occur if irreversible changes have occurred at the neuromuscular junction [8]; (3) For mixed nerves, issues such as misdirected reinnervation can arise [9].

On the other hand, recent studies have demonstrated the effectiveness of nerve conduits for motor nerves in animal models, suggesting a potential alternative to autologous nerve transplantation [10,11].

In our case, the gap between the nerve ends was less than 3 cm in length, and the injured posterior interosseous nerve was relatively thin. As this was a predominantly motor nerve, the risk of misdirected reinnervation was low, and the injury site was relatively close to the neuromuscular junction, which contributed to a relatively good recovery following nerve conduit repair. Although the use of a nerve conduit for motor nerves may not be as effective as autologous nerve grafts, nerve conduits could be a viable option for motor nerve injuries involving small gaps and thinner nerves, especially when the injury is near the neuromuscular junction.

In this case, the recovery of the thumb function was poor. This may be due to the distal end of the radial side of the damaged nerve being shorter and more severely damaged than the ulnar side. Additionally, if the separated nerve fascicles had been sutured with nerve conduits individually, the recovery might have been better. This is something that will need further consideration as we accumulate more cases in the future.

## Conclusions

In this study, we successfully utilized a nerve conduit for the reconstruction of motor nerve defects. The patients demonstrated significant functional recovery, with restored voluntary movement and electrophysiological improvement observed within five months postoperatively. These findings suggest that the nerve conduit may serve as one of the treatment options for motor nerve repair. Further clinical studies are needed to validate its long-term efficacy and broader applications.
